# Role of Calcitriol and Vitamin D Receptor (*VDR*) Gene Polymorphisms in Alzheimer’s Disease

**DOI:** 10.3390/ijms25094806

**Published:** 2024-04-28

**Authors:** Soon Pyo Jeong, Niti Sharma, Seong Soo A. An

**Affiliations:** Bionano Research Institute, Gachon University, 1342 Seongnam-daero, Sujung-gu, Seongnam-si 461-701, Republic of Korea

**Keywords:** vitamin D, calcitriol, *VDR* polymorphism, Alzheimer’s diseases, neuroprotection

## Abstract

Alzheimer’s disease (AD) is characterized by amyloid beta (Aβ) buildup and neuronal degeneration. An association between low serum vitamin D levels and an increased risk of AD has been reported in several epidemiological studies. Calcitriol (1,25-dihydroxycholecalciferol) is the active form of vitamin D, and is generated in the kidney and many other tissues/organs, including the brain. It is a steroid hormone that regulates important functions like calcium/phosphorous levels, bone mineralization, and immunomodulation, indicating its broader systemic significance. In addition, calcitriol confers neuroprotection by mitigating oxidative stress and neuroinflammation, promoting the clearance of Aβ, myelin formation, neurogenesis, neurotransmission, and autophagy. The receptors to which calcitriol binds (vitamin D receptors; VDRs) to exert its effects are distributed over many organs and tissues, representing other significant roles of calcitriol beyond sustaining bone health. The biological effects of calcitriol are manifested through genomic (classical) and non-genomic actions through different pathways. The first is a slow genomic effect involving nuclear VDR directly affecting gene transcription. The association of AD with *VDR* gene polymorphisms relies on the changes in vitamin D consumption, which lowers *VDR* expression, protein stability, and binding affinity. It leads to the altered expression of genes involved in the neuroprotective effects of calcitriol. This review summarizes the neuroprotective mechanism of calcitriol and the role of *VDR* polymorphisms in AD, and might help develop potential therapeutic strategies and markers for AD in the future.

## 1. Introduction

Alzheimer’s disease (AD) is the most prevalent neurodegenerative disease (ND) that results in progressive damage to the structure and function of neurons. Globally, over 400 million people are affected by AD and it is the main cause of dementia in the elderly population [1]. The pathogenesis of AD is multifactorial and is associated with the deposition of amyloid-β (Aβ) plaques and neurofibrillary tangles (NFTs), neuronal loss accompanied with increased oxidative stress, neuroinflammation, and synaptic changes [2,3]. The available Food and Drug Administration (FDA)-approved drugs (Donepezil, Galantamine, Rivastigmine, Memantine, Aduhelm) provide only symptomatic treatment [4] but a treatment to prevent the disease is lacking. The development of an effective, safe, and low-cost treatment would undoubtedly have a great global impact.

Various studies have shown an association of vitamin D deficiency with an increased risk of infections, including COVID-19; asthma; cardiovascular diseases; autoimmune diseases; hyperlipidemia; diabetes; and cancer [5,6,7,8,9]. The vitamin D status is evaluated from serum calcifediol levels (longer half-life compared to other vitamin D metabolites) and a value > 30 ng/mL is considered to be healthy. The recommended dietary allowance (RDA) of vitamin D for different age groups is 400 IU (0–12 months); 600 IU (up to 70 years), and 800 IU (over 70 years) [10]. Calcitriol, the bioactive form of vitamin D, is a fat-soluble vitamin with antioxidant activity, maintains calcium and phosphorous homeostasis, and strengthens immune function. Studies indicate a novel link between calcitriol and mitochondrial bioenergetics involving calcium channels [11]. Calcitriol displays its effect by binding to vitamin D receptors (VDRs), which are present in various tissues and organs such as the skin, brain, parathyroid, skeletal muscles, heart muscles, pancreas, pituitary, ovaries, testes, and blood cells, but not the kidney and bones [12]. The diverse locations of VDRs indicate other important functions of calcitriol in the body. The research in the past two decades has identified calcitriol as a likely neurosteroid [13] and its possible link with psychiatric disorders and [14,15] NDs was explored [16,17,18]. The brain is capable of synthesizing and receiving calcitriol, which is speculated to support synaptic plasticity and neurotransmission [19,20], and its role in neurogenesis, preventing neuroinflammation, improving cognition, and the clearance of amyloid plaques has been studied [21,22,23,24]. It is also known to protect the CNS from immunopathogenic diseases and inflammasome activation. Early intervention with calcitriol ameliorates the activation of local microglia/macrophage, preventing neuroinflammation [24]. Calcitriol improves Aβ clearance by stimulating phagocytosis along with reduced monoamine oxidase B (MAO-B) expression [25]. Calcitriol is known to be present after brain injury and protects the integrity of the blood–brain barrier (BBB) following acute ischemic stroke [26]. Calcitriol possesses good antioxidant activity, reduces cell apoptosis, and increases the production of neurotrophic factors [nerve growth factors (NGFs) and glial cell line derived neurotrophic factors (GDNFs), neurotrophin-3, Brain-derived neurotrophic factors (BDNFs)] required for the survival and growth of neurons, which ultimately improves cognition [26,27].

In the present review, we provide an overview of the neuroprotective mechanism of calcitriol and *VDR* polymorphisms in AD. Understanding the molecular mechanisms and the role of genetic variation could be important for developing therapies for NDs in the future.

## 2. Methods

The present work gives an inclusive overview of the published scientific research available on various databases (PubMed, Google Scholar, and Science Direct) up until January 2024. The search terms used were “calcitriol” or “vitamin D” or “vitamin D receptor” or “vitamin D transport” with the filter “brain”, “neuroprotection”, “Alzheimer’s disease”, “*VDR* polymorphism”, and “English”. Exclusion criteria: the papers not in the English language were not included.

## 3. Synthesis and Activation of Vitamin D

**Figure 1:**
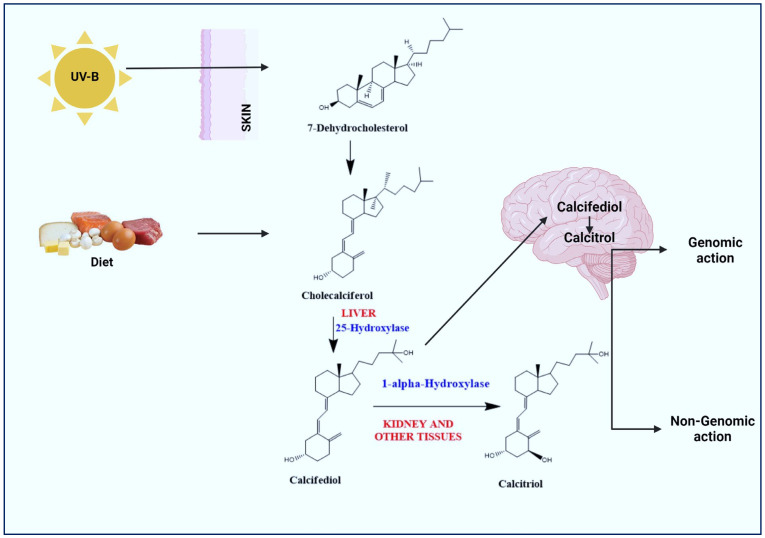
Activation of vitamin D to calcitriol. Exposure of skin to sunlight (UV-B) results in conversion of pro-vitamin D_3_ (7-dehydrocholesterol) to pre-vitamin D_3_ (Cholecalciferol). Diet/vitamin D supplements bind to vitamin D-binding proteins and are transported to liver and hydroxylated by 25-Hydroxylase to calcifediol. In the kidneys and other tissues/organs including the brain, calcifediol is hydroxylated to calcitriol (active form of vitamin D) by 1α-hydroxylase. Calcitriol affects different targets through genomic and non-genomic pathways.

Vitamin D prohormone (calciferol; sunshine vitamin) is a collective term for vitamin D_2_ (ergocalciferol) and vitamin D_3_ (cholecalciferol). Vitamin D_3_ is mainly synthesized in the skin through the photochemical action of solar ultraviolet type B (UVB) radiation on 7-dehydrocholesterol (7-DHC) (Figure 1). In contrast, the body is unable to produce vitamin D_2_ and can only be consumed through the consumption of plants in the diet. Vitamin D_2_ has a double bond between C_22_ and C_23_ and a methyl group on C_24_, which is absent in vitamin D_3_. This structural difference affects metabolic activity and thus vitamin D_3_ exhibits a better affinity for vitamin D binding protein (VDBP) and vitamin D receptor (VDR) [28]. Vitamin D is biologically inert and requires two hydroxylation steps for its activation. Initially, it is hydroxylated in the liver by cytochrome P450 enzyme (CYP2R1; 25-Hydroxylase) to calcifediol (25-hydroxy vitamin D_3_; [25(OH)D_3_]) and further metabolized to calcitriol (1,25-dihydroxy vitamin D_3_; 1,25(OH)_2_D_3_) by CYP27B1 (1α-Hydroxylase) in the kidneys and other tissues/organs, including the brain.

## 4. Transport of Vitamin D

The major circulating form of vitamin D is serum calcifediol (half-life of 15–20 days), which is in equilibrium with the level of vitamin D stored in muscle and adipose tissues. The plasma or serum calcifediol concentration is inversely related to the risk of vascular dementia and AD in the elderly [29]. Patients with calcifediol (<10 ng/mL) tend to score lower in the Mini-Mental State Examination (MMSE) and Montreal Cognitive Assessment (MoCA), and have an increased risk of developing cognitive impairment calculated by the Clinical Dementia Rating (CDR) scale [30]. A meta-analysis on cohort studies using the prediction interval (PI) to describe the heterogeneity described an inverse relation between the calcifediol level and the risk of dementia and AD, consistent with a linear dose–response relationship. Additionally, the data indicated a decrease in dementia (5%) and AD (7%) with each increase of 10 nmol/L of calcifediol [31]. In the past, few other similar studies have shown an inverse relation between AD and low calcifediol but a causal association could not be established either due to the small sample groups and the method of quantifying the heterogeneity or restricting the categories of the calcifediol level [32,33,34].

As a result, the serum calcifediol is considered a marker to evaluate the vitamin D status in the body. Albumin helps in the transport of some vitamin D metabolites but the majority (~85%) are transported to various tissues by binding to VDBP, and among them, calcifediol has a stronger binding affinity to VDBP compared to the others [35]. When the levels of vitamin D exceed the tolerated levels in the body, it is metabolized by CYP24A1 to inactive metabolites, i.e., 1,24,25(OH)D_3_, calcitroic acid, and 24,25(OH)_2_D_3_. Bioavailable vitamin D is the sum of the free and albumin-bound forms, which constitute around 15% in a healthy person [36]. VDBP is a highly polymorphic serum α2-globulin (52–59 kDa) widely distributed in various tissues. It performs diverse functions besides binding to vitamin D, like scavenging endotoxins and actin, binding fatty acids, and mediating the immune system [37]. The VDBP levels were reported to be altered in disease conditions [38,39,40] that affect the total level of vitamin D.

For most cells, it is the unbound calcifediol that enters cells (free hormone hypothe-sis); however, in some cases (kidney, parathyroid gland, and placenta), VDBP-bound calcifediol is transported in the cell via a megalin/cubilin complex [41]. An association of single nucleotide polymorphisms (SNPs) of megalin and VDBP with AD and PD has been reported, respectively [42,43].

## 5. Distribution of VDR in Brain

VDR belongs to the zinc finger steroid hormone nuclear receptor family and is widely distributed in the body [44]. After Sutherland et al. [45] reported the expression of *VDR* in AD brains, various studies reported *VDR* expression in neuroblastoma cell lines [46] and in the developing and adult brain in various mammalian species [47,48,49]. The increased expression of *VDR* with gestational age indicates the importance of vitamin D in maintaining normal brain development [47]. Vitamin D deficiency in pregnancy has been associated with various neurological issues like autism, attention deficit hyperactivity disorder (ADHD), and schizophrenia in infants [15,50,51,52,53]. Studies have established the presence of VDR and CYP27B1 in neurons and glial cells and CYP24A1 in astrocytes was established [13]. In the hippocampus, VDR is present exclusively in the CA1 and CA2 regions while a minor amount is present in CA3. VDR is concentrated in the nucleus whereas CYP27B1 is dispersed all over the cytoplasm. Both VDR and CYP27B1 are present in equivalent amounts in most of the brain regions, with the maximum amount reported in the hypothalamus and substantia nigra. However, CYP27B1 is exclusively present in Purkinje neurons (cerebellum) and substantia innominata neurons (basal forebrain) [13].

The key forms of vitamin D [calcifediol, calcitriol and 24,25(OH)_2_D_3_] have the ability to cross the BBB and exist in human cerebrospinal fluid (CSF) [54]. Moreover, the enzymes for synthesizing (CYP27B1) and catabolizing (CYP24A1) calcifediol are present in the brain, suggesting in situ synthesis of calcitriol and autoregulated elimination in the brain. The calcitriol levels in the brain correlate with the plasma calcifediol [55] and remain unaffected by vitamin D supplementation, again proving the local synthesis of calcitriol in the brain [56]. Recently, the Ultra-Pressure LC-Tandem Mass Spectra (UPLC-MS) technique was used to evaluate vitamin D metabolites in the human brain. All the examined regions contained calcifediol, with the corpus callosum being the richest in calcifediol (334 pg/g). On the other hand, low levels of calcitriol were spotted in the prefrontal (30 pg/g) and middle frontal cortices (35 pg/g) only [57].

## 6. Molecular Mechanism of Action of Calcitriol

**Figure 2:**
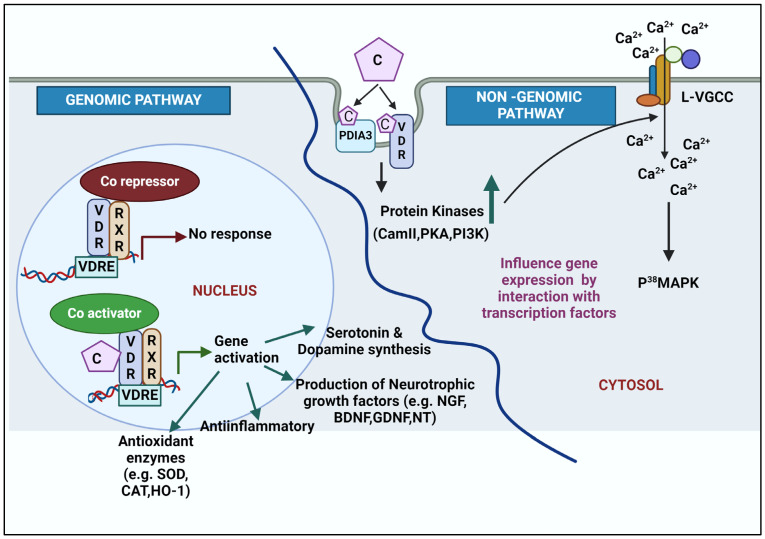
A schematic for genomic and non-genomic action of calcitriol in brain. Genomic pathways occur in the nucleus. Binding of calcitriol to the VDR/RXR complex removes the co-repressors and interacts with co-activators on the VDREs located on regulatory regions to promote gene expression. The non-genomic action takes place in the cytosol and is initiated by the binding of calcitriol to VDR, PDIA3, or both. The binding activates protein kinases, which facilitates influx of calcium through L-VGCC. Intracellular calcium activates p38MAPK to further modulate downstream signaling. Abbreviations: VDR: vitamin D receptor; RXR: retinoic acid receptor; VDRE: vitamin D response element; L-VGCC: L-type voltage-gated calcium channel; PDIA3: protein disulphide isomerase family member 3; CaM II: Ca^2+^/calmodulin-dependent protein kinase; PKA: protein kinase A; PI3K: phosphatidylinositol-3-kinase, p38MAPK: mitogen-associated kinase; C: calcitriol; Ca^2+^: calcium; SOD: superoxide dismutase; CAT: catalase; HO-1: heme oxygenase; NGF: neurotrophic growth factors; BDNF: brain-derived neurotrophic factor; GDNF: glial cell-derived neurotrophic factor; NT: neurotrophin.

The active form of vitamin D is calcitriol, which has a crucial role in brain development and neuroprotection. Calcitriol exerts its action by binding to VDR through genomic (classical) pathways (slow response) or non-genomic pathways (rapid response) (Figure 2).

### 6.1. Genomic Action of Calcitriol

The endocrine action of calcitriol is mediated through VDR. Calcitriol has a high binding affinity (~1000 times) for VDR as compared to calcifediol [58] due to the interactions of its three hydroxyl groups with polar amino acids in the ligand binding pocket. Yet, the 1000-fold higher serum concentrations of calcifediol (50–250 nM) compared to calcitriol compensate for its effective binding to VDR [59].

The binding of calcitriol to the VDR leads to the formation of a heterodimeric complex with retinoic acid X receptor (RXR). The protein importin-β, and its ligand, vitamin A, help in the transport of RXR to the nucleus, while importin-α assists with the transport of VDR to the nucleus, and this process is significantly improved by the mediation of calcitriol. The complex is then transported into the nucleus where it binds to vitamin D response elements (VDREs) [60] with the release of co-repressors (nuclear receptor co-repressor 2/silencing mediator of retinoic acid and thyroid hormone receptor: NcoR2/SMART) and recruitment of coactivators, which increase histone acetylation (steroid receptor co-activator: SRC1) and modify chromatin (lysine demethylase 6B) to promote the expression of several genes involved in bone calcium homeostasis [alkaline phosphatase (*ALP*), osteopontin (*SPP1*), stanniocalcin 1 (*STC1*), Transient receptor potential vanilloid type 6 (*TRPV6*)], parathyroid hormone (PTH), vitamin D hydroxylases (CYP27A, CYP27B1, CYP24), calcitriol-responsive endobiotic/xenobiotic metabolizing enzyme (CYP3A4), and metallothionein 2 (MT2) [61,62,63]. Such activated genes may further modulate the action of other genes as a secondary genomic response [64]. Genomic regulation has epigenetic effects as it modifies the expression of enzymes involved in methylation and acetylation [65]. Additionally, micro-RNA (miR) expression that controls post-transcriptional gene expression and silencing is also controlled by this mechanism [66]. In short, calcitriol, VDREs, and modulators bound to the heterodimer-RXR are responsible for the physiological, genetic, and cell/tissue specificities, respectively. Lastly, the *VDR* gene product exerts a biological response [67].

In the brain, calcitriol has been reported to exert multifaceted neuroprotection by increasing the expression of glial-derived neurotrophic factor (GDNF) in the cortex [68] and striatum [69], and the enzyme for the synthesis of dopamine, i.e., tyrosine hydroxylase (TH), and N-cadherin (neural adhesion molecule with role in neurogenesis), which in turn increase dopaminergic (DA) neurons [70,71]. To maintain ideal neurotransmission, DA neurons stimulate the expression of DA-catabolizing enzymes (catechol-O-methyltransferase: COMT and monoamine oxidase A (MAO_A_) by negative feedback [72]. Hence, calcitriol could be a promising therapy in Parkinson’s disease (PD). Calcitriol was reported to increase serotonin neuronal cells by increasing the expression of the enzyme (tryptophan hydroxylase-2: TPH2) involved in its synthesis, and suppressing the enzyme involved in its catabolism (MAO_A_) and the serotonin reuptake transporter (SERT) [73]. Calcitriol also downregulated MerTK expression [74], reducing the phagocytosis of myelin and apoptotic T cells, which is beneficial in the treatment of multiple sclerosis. Amyloid beta has been known to overturn the expression of *VDR* in cortical neurons by modulating nerve growth factor (NGF) synthesis and Ca^2+^ homeostasis [75].

### 6.2. Non-Genomic Action of Calcitriol

The non-genomic pathways are rapid compared to the genomic response and are mediated through specific membrane receptors like VDR and PDIA3 (protein disulfide isomerase family A member 3) (Figure 2). VDR interacts with various target proteins such as β-catenin, c-Jun, STAT1, inhibitor of nuclear factor-κB (IκB) kinase (IKK), cAMP response element-binding protein, and Runt-related transcription factor 1 (RunX 1) in the non-genomic response in vitro [76,77].

The regulation of Ca^2+^ channels, activation of protein kinases (protein kinase A: PKA; protein kinase C: PKC; mitogen-activated protein kinase: MAPK; Phosphoinositide 3- kinase: PI3K; Ca^2+^/calmodulin-dependent kinase II: CaMKII) and phospholipase (Phospholipase A2: PLA2; Phospholipase C; PLC) are some of the non-genomic signal transduction events triggered by calcitriol. These actions do not require gene transcription. Additionally, calcitriol, via the non-genomic pathway, indirectly influences the expression of various genes by interacting with multiple transcription factors (like Aryl hydrocarbon Receptor: AhR; Nuclear factor kappa: NF-κB; Nuclear factor erythroid-2-related factor 2: Nrf2, RAR-related orphan receptor alpha and gamma: RORα, RORγ; Signal transducer and activator of transcription 3: STAT3) [78].

In the microglia, calcitriol exerts the neuroinflammatory response mostly through a non-genomic mechanism by inhibiting the translocation of NF-κB and phosphorylation of extracellular signal-related kinase (ERK) [79]. Calcitriol and calcifediol also upregulate the expression of the anti-inflammatory cytokine (IL-10), which in turn induces suppressor of cytokine signaling (SOCS) to downregulate pro-inflammatory cytokines [80]. Calcitriol supplementation promoted cell proliferation and reduced senescence in [1α(OH)ase^−/−^] mice by upregulating Nrf2, reducing oxidative stress, and inactivating senescence-promoting genes (p^53^/p^21^ and p^16^/p^Rb^) [81].

## 7. Genetic Variants in *VDR* and Risk of Late-Onset AD (LOAD)

The apolipoprotein E (*APOE*) gene, present on chromosome 19, has been recognized as the major risk gene for LOAD, as ~50% of patients diagnosed with LOAD have the *APOEε4* gene [82]. This gene causes neurodegeneration by affecting the neuronal cytoskeleton, inhibiting neurite outgrowth, and stimulating tau phosphorylation [83]. Other genes, related to cholesterol biosynthesis (*CLU*, *ABCA7*), endocytosis, synaptic function (*CD2AP*, *BIN1*, *PICALM*), and inflammation and the immune response (*CR1*, *CD33*, *EPHA1*, *TREM2*, *MS4A*) have been identified through genome-wide association studies (GWASs) [84].

In the search for additional genetic risk factors for LOAD, studies have reported the association of *VDR* gene polymorphisms and cognitive decline in NDs [85,86,87]. The *VDR* gene (100 kb) is located on chromosome 12 (12q13–12q14) and consists of nine exons and eight introns. Exons 2 and 3 encode the DNA binding site, while exons 4 to 9 encode the ligand binding site. This locus was reported to be a vulnerable locus linked to LOAD [88]. According to GWASs, *VDR* is among the most likely risk genes for developing AD [89]. The association of AD with *VDR* gene polymorphisms suggests a potential negative impact on the neuroprotective effect of calcitriol. Changes in vitamin D consumption are said to lower *VDR* expression, protein stability, and binding affinity, leading to the altered expression of genes involved in neuroprotection.

**Figure 3:**
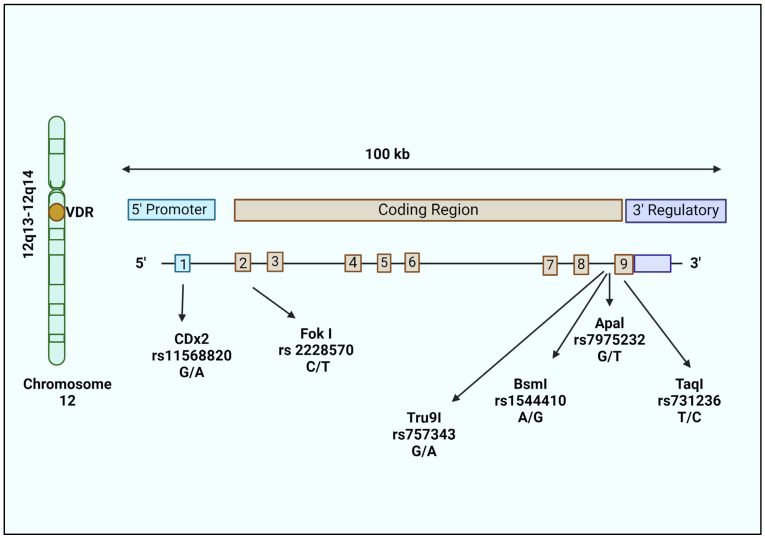
Structure of the *VDR* gene and position of polymorphisms studied in AD. The *VDR* gene is located on chromosome 12 (12q13–12q14). The *VDR* gene is 100 kb and divided into 8 introns and 9 exons. The first exon contains the gene promoter, exon 2–3 code for the DNA binding domain, and exon 6–9 for the ligand binding domain. Widely studied polymorphisms in AD include Fok-I (rs10735810), Apa-I (rs7975232), Bsm-I (rs1544410), Taq-I (rs731236), and Tru9I (rs757343).

Several single nucleotide polymorphisms (SNPs) have been identified in the *VDR* gene with roles in various disease conditions. But *VDR* gene polymorphisms in most cases do not affect VDR function, as they do not result in an amino acid change. The main *VDR* SNPs studied in relation to AD are ApaI, TaqI, BsmI, and FokI, which lead to changes in vitamin D utilization, leading to increased neurodegeneration [90]. ApaI (rs7975232; intron 8; G to T polymorphism), TaqI (rs731236; exon 9; T to C polymorphism), and BsmI (rs1544410; intron 8; A to G polymorphism) are 3′ UTR polymorphisms that increase mRNA stability. The FokI (rs2228570; exon 2; C to T polymorphism) polymorphism alters the gene’s initiation sites and modifies the protein’s structure by extending its length by three amino acids. As a result, two VDR variants exist, and the long version of VDR is called M1 (methionine at first position; T-allele or the f allele), while the short form is referred to as M4 (methionine at fourth position; C-allele or F allele). The Cdx2 (rs11568820; promoter region; A to G polymorphism) polymorphism might affect the transcriptional activity [91], whereas Tru9I (rs757343; intron 8; G to A polymorphism) is associated with serum vitamin D levels [92] (Figure 3).

In the aged population (over 75 years old) Apa1 polymorphisms (T allele) and Taq1 polymorphisms (G allele), were found to be associated with AD and also displayed interactions with genes regulating inflammation (interleukin 10: IL-10; dopamine-β hydroxylase: DBH) [93]. The ApaI polymorphism (A allele) was associated with a 30% lower risk of AD in Polish and British populations [94], while only the TaqI polymorphism (T allele) was significantly associated with the risk of AD in a Korean population. However, in an Iranian population, the ApaI and TaqI polymorphisms were not associated with the risk of LOAD [95]. ApaI (C allele) and TaqI (T allele) were more frequent in individuals with mild cognitive impairment (MCI) in a Chilean population. In addition, these polymorphisms decreased the expression of p-glycoprotein (p-gp), the transporter of β-amyloid peptide (Aβ) [96]. Another contrasting result was obtained for the TaqI C allele, which increased the AD risk in Northwestern European Caucasians (NECs) [93], but exerted a protective effect in a Southeastern European Caucasian (SEC) population by decreasing the AD risk by 46% [97], and caused no effect in an elderly Brazilian population [98]. Another study’s results showed that the TaqI polymorphism (dominant and homozygous) and BsmI (recessive) and FokI polymorphisms (heterozygous) were linked with increased AD risk in Caucasian and Asian populations, respectively [87]. A recent study provided a statistical indication that the ApaI and BsmI polymorphisms were connected to a risk of MCI, while the TaqI polymorphisms may have been associated with AD risk [87]. In another study, the risk allele of CDX2 lowered *VDR* promoter activity in Neuro2A cells [99] and a strong association between SNP CDX2 and an LOAD patient group was observed. The contradictory results obtained from different populations indicate that both genetic and non-genetic factors such as ethnicity, climate, and environment modify the expression and response of VDR [87]. This specifies that external factors impacting vitamin D intake could potentially compromise the efficacy of its neuroprotective mechanism.

The studies to identify a potential link between *VDR* SNP haplotypes are limited. Strong linkage disequilibrium (LD) was observed in BsmI, ApaI, and TaqI polymorphisms that described five haplotypes (Table 1). The frequency of haplotype 1 (CCA) and haplotype 2 (TAG) was the highest (45.5% and 42.4%, respectively) of the other three in an elderly (over 85 years) population [85]. Gezen-Ak et al. [89] found no significant difference between AD vs. controls when the BsmI, Tru9I, and FokI polymorphic sites were compared. A haplotype analysis carried out with additional SNPs indicated a significantly higher frequency of the “TaubF” haplotype (corresponds to alleles of TaqI, ApaI, Tru9I, BsmI, and FokI, respectively) in the AD patient group, suggesting that this haplotype is a risk factor for AD. The BsmI, ApaI, and TaqI polymorphisms, located in the 3′-UTR, were identified as risk haplotypes associated with age-related cognitive decline [85]. In yet another cohort study on SECs, the TAC (TaqI, BsmI, FokI) and TA (TaqI, BsmI) haplotypes were associated with a nearly 6% and 8% increase in the LOAD risk, respectively. On the other hand, the CAC (TaqI, BsmI and FokI) haplotype was protective with a 53% lesser risk of developing AD. Additionally, SEC female TAC/TC carriers carry a greater risk (approximately 9 to 14%) of developing AD, suggesting that this haplotype affects vitamin D utilization [97].

## 8. Conclusions

Various in vitro, in vivo, and epidemiological studies have established that vitamin D (calcitriol) is required for healthy brain function and its deficiency during pregnancy might result in several neurological conditions in newborns. The association of circulating vitamin D levels and AD risk has been proven by Mendelian randomization studies in large datasets [100], which provide a potential avenue for understanding and preventing the disease. In AD, calcitriol exerts multi-targeted neuroprotection via both genomic and non-genomic actions, resulting in Aβ clearance, the promotion of neurogenesis and production of neurotrophic factors, a decrease in neuroinflammation and oxidative stress, the downregulation of the expression of L-type calcium channels, and improved cognition [25,101,102]. It is possible that calcitriol’s therapeutic effects will take longer to manifest because of the slow effects of the genomic pathways; thus, it could be used as a preventive approach in AD. Nevertheless, controversial outcomes have also been reported in some studies [103,104,105]. The contradictory results might stem from diverse dosages, the duration of treatment, the age/sex of the patient, the population size, and the vitamin D level of each patient under study. Thus, clinical trials need to be planned intricately, considering these variables.

Vitamin D from foods and exposure to sunlight is not adequate in the majority of cases, and hence, vitamin supplementation is required. Studies have suggested a positive effect of vitamin D supplementation, especially in people with severe vitamin D deficiency compared to vitamin D-replete people, and that a vitamin D intake ≥ 4000 IU/day conveys some health risks [106]. Clinical trials on vitamin D supplementation and cognition have resulted in conflicting conclusions [107,108]. Clinical trials have varied in terms of the formulation (cholecalciferol/ergocalciferol/calcium–vitamin D) of vitamin D [109,110,111]. Cholecalciferol supplementation was found to be more effective [112] and the use of calcium in the formulation increased intestinal calcium absorption [113]. Moreover, it is important to study whether low serum calcifediol is the result of the disease or is causing the disease.

The *VDR* gene is regulated by genetic and environmental factors and the response to vitamin D supplementation could be affected by *VDR* polymorphisms, e.g., the FF genotype of VDR Fok I is associated with a better response to vitamin D supplementation [114]. Association studies on VDR polymorphisms and the risk of AD might be helpful in designing custom-made methods for treating NDs like AD. The VDR polymorphisms ApaI and BsmI predict MCI, while TaqI indicates a risk of AD, with population differences. As the vitamin D supplementation requirement varies based on the *VDR* polymorphism, this presents a possible limitation, as the effectiveness of the treatment may depend on genetic factors, potentially limiting its applicability across different individuals. Therefore, further research is needed to confirm these associations, particularly gene-gene interactions and gene-environment interactions, and interactions with other confounding factors should be considered.

## Figures and Tables

**Table 1:** *VDR* haplotypes associated with AD.

FokI	ApaI	BsmI	TaqI	Tru9I	Ref.
	C	C	A		[85]
C	C	G	T	A	[89]
	A		C		[93]
C		A	T		[97]
